# Plasmonic Structures, Materials and Lenses for Optical Lithography beyond the Diffraction Limit: A Review

**DOI:** 10.3390/mi7070118

**Published:** 2016-07-13

**Authors:** Changtao Wang, Wei Zhang, Zeyu Zhao, Yanqin Wang, Ping Gao, Yunfei Luo, Xiangang Luo

**Affiliations:** State Key Laboratory of Optical Technologies on Nano-fabrication and Micro-engineering, Institute of Optics and Electronics, Chinese Academy of Sciences, Chengdu 610209, China; wangct@ioe.ac.cn (C.W.); zhaocw@ioe.ac.cn (W.Z.); zhaozy@ioe.ac.cn (Z.Z.); wangyq@ioe.ac.cn (Y.W.); gaop@ioe.ac.cn (P.G.); luoyf@ioe.ac.cn (Y.L.)

**Keywords:** surface plasmon polaritons, bulk plasmon polaritons, diffraction limit, subwavelength optics, near-field optics, metamaterial, super resolution, nano optical lithography, nanostructure fabrication

## Abstract

The rapid development of nanotechnologies and sciences has led to the great demand for novel lithography methods allowing large area, low cost and high resolution nano fabrications. Characterized by unique sub-diffraction optical features like propagation with an ultra-short wavelength and great field enhancement in subwavelength regions, surface plasmon polaritons (SPPs), including surface plasmon waves, bulk plasmon polaritons (BPPs) and localized surface plasmons (LSPs), have become potentially promising candidates for nano lithography. In this paper, investigations into plasmonic lithography in the manner of point-to-point writing, interference and imaging were reviewed in detail. Theoretical simulations and experiments have demonstrated plasmonic lithography resolution far beyond the conventional diffraction limit, even with ultraviolet light sources and single exposure performances. Half-pitch resolution as high as 22 nm (~1/17 light wavelength) was observed in plasmonic lens imaging lithography. Moreover, not only the overview of state-of-the-art results, but also the physics behind them and future research suggestions are discussed as well.

## 1. Introduction

Since its invention in about 1960, optical lithography has made a large contribution to the present integrated circuits industry, and has been widely employed in investigations spanning electronics, micro optics, photonics, medical and biology, new materials, etc. The diffraction-limited resolution of optical lithography, usually referred to as about half the wavelength of light, hampers its applications for nano patterns’ fabrication. To solve this problem, light sources with a shorter wavelength are employed in advanced optical lithography, including deep ultraviolet (DUV) of 248 and 193 nm, and even extreme ultraviolet (EUV) of about 13.5 nm [1]. This, unfortunately, requires considerable complex optics, new materials and some other challenging technology problems, and presents a great increase in cost for users and researchers, especially for modest investigations [2]. Nowadays, researchers are reluctantly forced to use high-cost and point-to-point scanning writers, like electron beam lithography (EBL) [3,4] and focused ion beams (FIB) [5,6]. Thus, low-cost, high-efficiency and large-area nanolithography methods are preferred from the viewing point of practical applications.

Some novel techniques have been proposed and developed for improved resolution beyond the diffraction limit of conventional optical lithography. For instance, double-exposure techniques were proposed to get doubled dense nano patterns, but with increased expense, complexity and limited patterns [1]. Nano imprinting has been widely explored as a low-cost replica method of nano patterns [7,8,9,10]. Near-field lithography deserves special attention due to its inherent relation to the topic of this paper, in which evanescent waves diffracted from subwavelength mask patterns are employed to optically print nano patterns to a photoresist layer in very close proximity and even in a contacting manner [11,12,13]. This method seems to possess nearly unlimited resolving power in contacting mode but suffers from ultra-short exposure depth and poor fidelity, etc. [13]. Also, some other proposed novel methods show promising but limited nanofabrication ability for periodic patterns and specific materials, like directed self-assembling [14], dip-pen [15] and heat pens [16], etc.

Recently, the investigations of and physics behind some abnormal optical phenomena associated with surface plasmon polaritons (SPPs), like extraordinary optical transmission through subwavelength metallic holes array [17], some proposed novel concepts like perfect lens [18] and metamaterial, inspired us and some other research teams to be aimed at plasmonic lithography. The intriguing features of SPPs, including much shorter propagation wavelength than that of light in a dielectric medium with equal frequency and great field enhancement and energy concentration within a subwavelength region, are the main reasons for taking SPPs as a potential way of nanolithography beyond the diffraction limit. As an example, Luo performed subwavelength SPP lithograph experiments since 2003 [19,20], obtaining about 50 nm line-width patterns using SPPs interference and a g-line mercury lamp of 436 nm [21]. Further, partly within the scheme of perfect lens, plasmonic lenses in a variety of forms like super lenses [22], plasmonic reflective lenses [23] and plasmonic cavity lenses [24] were designed and employed to realize imaging lithography with minimum half-pitch resolution down to 22 nm, about 1/17 light wavelength. Some nano devices like metalenses and nano polarizers have been fabricated by plasmonic imaging lens lithography. It is worth notifying that the short exposure depth issue in conventional near-field lithography could be considerably relived in this case, with the help of evanescent waves amplification and resolution-enhanced technologies by engineering the wave front of SPPs in the imaging process [25]. This point is very important from the viewpoint of practical applications, as separating the operational manner could be supported in plasmonic imaging lens lithography. Also, some plasmonic nano focusing structures for converging SPPs energy to a subwavelength point, like localized surface plasmons (LSPs) holes [26], SPP focusing structures with concentric rings [27] and bowties [28], are proposed and successfully demonstrated for point-to-point scanning nano lithography. For plasmonic lithography methods, nano patterns could be fabricated by only employing low-cost, long-wavelength light sources like mercury lamps and He-Cd lasers, etc., fully commercialized and developed materials of resists and no complex optics, just simple illumination of plasmonic lithography structures. Despite some inconvenient operations such as near-field close proximity, it provides a potentially promising nanofabrication lithography method characterized by low-cost, large-area advantages, and SPP imaging and interference lithography is believed to deliver much higher throughput than some conventional nano fabrication tools like EBL and FIB.

In this paper, we would like to review the research achievements of plasmonic lithography in the manners of direct writing, interference and imaging. Some representative and important results will be described in details, such as SPPs resonance interference lithography, bulk plasmon polaritons (BPPs) interference lithography, superlens lithography, plasmonic cavity lens lithography, and bowtie lithography, etc. Also discussed are the key aspects in evaluating the performance of plasmonic lithography, such as resolution, fidelity and the aspect ratio of nano patterns. Some new physics and materials accompanying plasmonic devices design and improvements of lithography will be presented as well, including structured light illumination with BPPs, two SPPs absorption and Fano resonance. The remaining problems and outlooks of further investigations of plasmonic lithography will be given in the end.

## 2. Characterizations and Excitations of Surface Plasmons

### 2.1. SPPs with Sub-Diffraction Features

**Figure 1:**
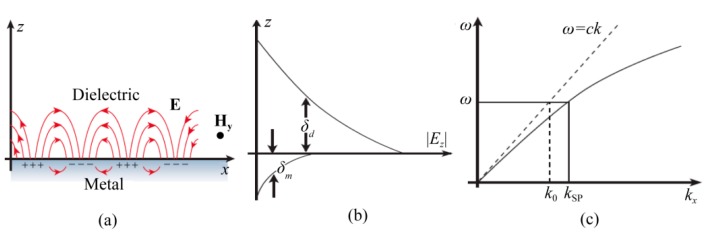
(**a**) SPPs (surface plasmon polaritons) on a dielectric–metal interface (*H* is in the *y* direction); (**b**) Field distribution of SPPs in the dielectric and metal material, *δ_d_* and *δ_m_* is the decay length of the field in the dielectric and metal material, respectively; (**c**) Dispersion curve for an SPP mode. Reproduced from [29]. Copyright © 2003, NPG.

SPPs exist around the interface between two mediums, one of which has free electrons or charges and the other does not, as shown in Figure 1a. SPPs are formed as electromagnetic field waves to excite free charges density oscillation and propagate along interfaces. To view it in a simple way, the charge density is usually ignored in the design and analysis of plasmonic structures by just considering medium with negative permittivity, which is usually described as the Drude model. In this way, Figure 1b indicates that SPPs are regarded as a special type of surface electromagnetic wave confined at the interface of dielectric and metal.

As a simplified and important case, the dispersion relationship of SPPs at the interface between two semi-infinite mediums takes the equation form below [29]:

(1) $$k_{\text{SP}}=\frac{\omega }{c}\sqrt{\frac{\varepsilon_{d}\varepsilon_{m}}{\varepsilon_{d}+\varepsilon_{m}}}$$

where *ω* is the angular frequency of light, *c* is light velocity in vacuum, *ε_m_* and *ε_d_* are the permittivity of metal and dielectric, respectively. Clearly, SPPs are slow waves and their dispersion relation is located at the right side of the light line in the diagram of transverse wavevector *k_x_* and angular frequency *ω* (Figure 1c). Thus, subwavelength interference patterns at the surface where SPPs are confined could be obtained, as two or multiple SPPs are launched and interfere with each other. It is worth noting that SPPs at planar surfaces show transverse magnetic mode polarization with one magnetic component *H_y_* normal to the SPPs propagation direction and two electric field components, *E_x_* and *E_z_*, with a complex magnitude ratio of ~*ik_z_*/*k_x_*. This would reduce the interference fringe contrast due to the π/2 phase shift between *E_x_* and *E_z_*.

**Figure 2:**
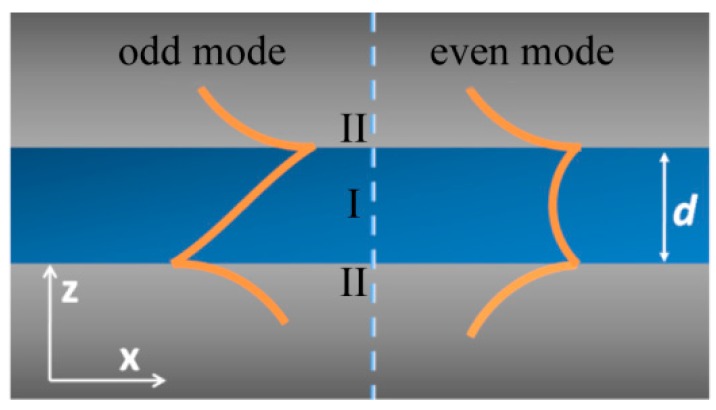
Schematic structure of metal–insulator–metal or insulator–metal–insulator (MIM or IMI) with *d* the thick core layer. The core layer and claddings are represented by Roman numbers “I” and “II”, respectively. The odd and even modes are represented by the orange-colored curve, respectively.

The semi-infinite SPPs mode could be viewed as an approximate case of thick metal film with a thickness much larger than the skin depth of SPPs inside the metal medium. In practical applications, two types of SPPs structures in Figure 2 are commonly encountered with a metal (dielectric) film sandwiched between two semi-infinite dielectric (metallic) mediums. This waveguide and cavity-like structure are usually used to tailor SPPs modes for modifying both transverse wave vector and electromagnetic field distribution. In these two modes, the SPPs dispersion relation equation is written in a similar form as [30]:

(2) $$\tanh k_{1}\frac{d}{2}=-\frac{k_{2}\varepsilon_{1}}{k_{1}\varepsilon_{2}}$$

(3) $$\tanh k_{1}\frac{d}{2}=-\frac{k_{1}\varepsilon_{2}}{k_{2}\varepsilon_{1}}$$

where *ε*_1_ and *ε*_2_ are permittivities of media I and II, respectively; $k_{1}^{2}=k_{x}^{2}-\varepsilon_{1}k_{0}^{2}$; $k_{2}^{2}=k_{x}^{2}-\varepsilon_{2}k_{0}^{2}$; *k_x_* is the transverse wave vector of SPPs wave.

SPPs in metal–insulation–metal (MIM) or insulation–metal–insulation (IMI) structure are split into two modes in comparison to that of metal–insulation (MI). From the view of wave coupling theory, this phenomenon could be explained by the coupling of two MI-SPP modes at the two interfaces of MIM or IMI structures. Usually, they are termed as odd and even modes according to the electric field or magnetic field distribution along the direction normal to the films. In some investigations, they are labeled with long-range and short-range SPPs to characterize the traveling length feature [31,32]. The difference mainly lies in the light confinement ratio modulation inside the metal medium with variant geometrical parameters, which help to reduce SPPs absorption due to metal loss. It is worth noting the magnitude difference of transverse and normal electric field components *E_x_* and *E_z_*, especially for the two SPPs modes inside the MIM structure. In the case of odd electric field distribution, the *E_z_* component dominates the SPPs field inside insulator region. For the even mode, however, the *E_x_* component magnitude is much larger than that of *E_z_*. This feature helps to get much higher contrast of imaging and interference fringes, compared with that of MI-SPP modes.

### 2.2. Excitation of SPPs

**Figure 3:**
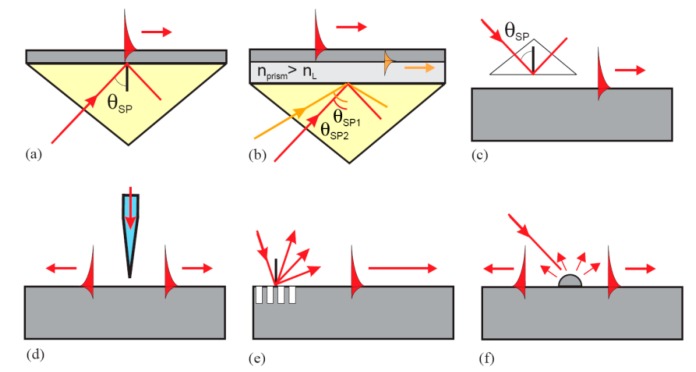
Typical configurations for SPPs excitation. (**a**) Kretschmann prism geometry, (**b**) Two-layer Kretschmann prism geometry; (**c**) Otto prism geometry; (**d**) Excitation with an SNOM probe; (**e**) Excitation by a grating, and (**f**) Excitation by surface features. Reproduced from [33]. Copyright © 2004, Elsevier.

At both sides of the meta–insulator interface, the electromagnetic fields drop exponentially, indicating that SPPs would not be excited just by impinging light on a smooth metal surface. To excite SPPs on a smooth planar surface, one needs to refer to some structures which could deliver extra *k_x_* beyond *k*_0_ and cover the *k_x_* gap between SPPs and the light line. Several structures are usually employed, including prism, grating, fiber tip and nano particle, which are presented in Figure 3. It is a key point to choose appropriate SPPs excitation methods for plasmonic lithography, considering the SPPs’ area size, high efficiency and complexity of operation. For instance, the three prism structures with Kretscmann form, double Kretscmann form and Otto form help to get large-area and high-efficiency SPPs modes excitations with proper configurations, but fail to excite SPPs with a much higher *k_x_*. On the other hand, some local small excitation structures like fiber tips, gratings and nano particles are able to get high *k_x_* SPPs, but show very limited SPPs mode region, as the SPPs get absorbed and scattered quickly as they go away from excitation regions.

### 2.3. BPPs of Hyperbolic Metamaterial

**Figure 4:**
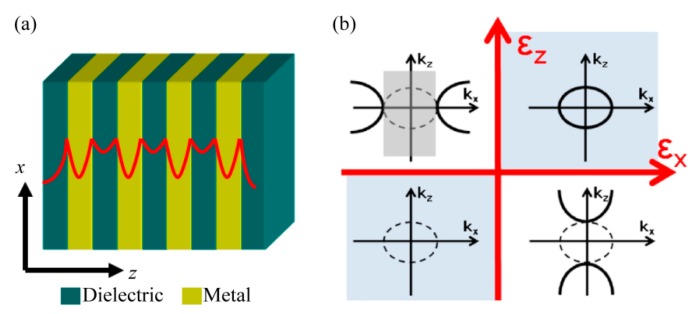
(**a**) Schematic of BPPs (plasmon polaritons) in hyperbolic metamaterial with alternative stacked metal–dielectric nano films; (**b**) Dispersion relation of the multilayer films with different signs of *ε_x_* and *ε_z_*. Reproduced from [34]. Copyright © 2014, AIP.

The term bulk plasmon polaritons (BPPs), as indicated by the word “bulk”, are the modes with electromagnetic field distribution across a volume space instead of being confined to and propagating along the interface of metal–dielectric media. As shown in Figure 4a, BPPs could be readily obtained in structures with alternatively stacked metal and dielectric films. In this configuration, each film thickness is small enough and SPPs are strongly coupled between adjacent films, yielding a whole unity of BPPs which exist and propagate in a 3D bulk space. In addition, the BPPs show some other abnormal features like directional light propagation [34,35], BPPs filter with specified wavevector range [36,37,38] and negative refraction behavior [39], etc., which plays an important role in designing plasmonic lithography structures, as shown below.

BPPs are investigated and analyzed numerically mainly in two ways, including effective medium theory (EMT) and rigorous coupled wave analysis (RCWA). In the first case, the multiple metallic and dielectric films are characterized with effective anisotropic permittivity in three directions as [40]:

(4) $$\varepsilon_{x}=\varepsilon_{y}=\varepsilon_{m}f+\varepsilon_{d}(1-f)$$

(5) $$\varepsilon_{z}^{-1}=\varepsilon_{m}^{-1}f+\varepsilon_{d}^{-1}(1-f)$$

where *ε_m_* and *ε_d_* are permittivity of metal and dielectric films, *f* is the filling factor of metal films thickness. Its equal-frequency contour (EFC) equation of dispersion relation in two-dimension space is expressed as [40]:

(6) $$\frac{k_{x}^{2}}{\varepsilon_{z}}+\frac{k_{z}^{2}}{\varepsilon_{x}}={\left(\frac{\omega }{c}\right)}^{2}$$

**Figure 5:**
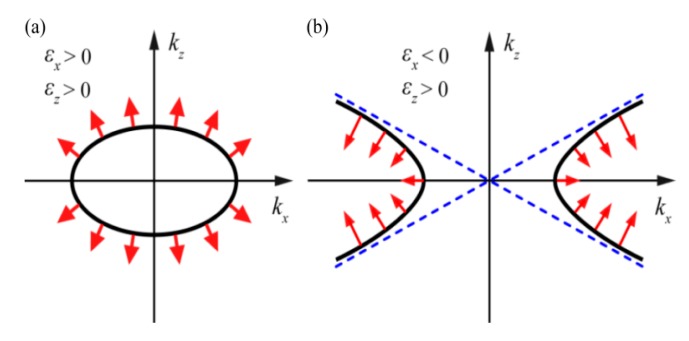
Hyperbolic equal-frequency contour (EFC) surface for metamaterial with (**a**) *ε_z_* > 0, *ε_x_* > 0; and (**b**) *ε_z_* > 0, *ε_x_* < 0. The group velocity is indicated by the arrows, which are perpendicular to the EFC.

For films with appropriate geometrical and permittivity parameters, the EFC is a hyperbolic function as shown in Figure 4b. In view of the dispersion relationship, some abnormal behaviors of BPPs inside hyperbolic metamaterial could be extracted [40,41,42]. For instance, evanescent waves with a large *k_x_* in dielectric medium become non-evanescent and propagating inside the hyperbolic systems. The abnormal BPPs directional propagation behaviors would be explained as normal to the hyperbolic EFC’s asymptotic curves (Figure 5). Also, spatial diffraction light through the films would be restricted to the desired *k_x_* wave vector ranges, where BPPs mode could be coupled out by the specific metamaterial. The spatial spectrum filtering behaviors could squeeze BPPs to form a large-area deep subwavelength interference pattern, which has been reported in many literatures [37,43,44].

Inside an absorption-free hyperbolic metamaterial, the EMT method shows spatial diffraction lights with infinite large transversal wave vector *k_x_* and *k_y_* the propagating modes, and the down limit of the cut-off wave vector is determined by $k_{x}^{2}+k_{y}^{2}=\varepsilon_{z}k_{0}^{2}$. However, the actual hyperbolic metamaterial processes the finite-thickness components with the substantial absorption, for example, five pairs of SiO_2_ and Al at the wavelength of 365 nm.

**Figure 6:**
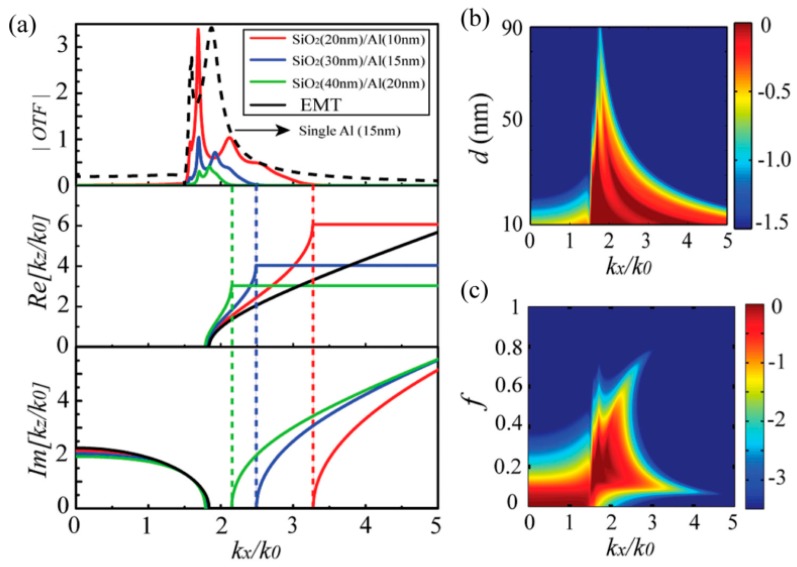
(**a**) Top panel is the calculation of optical transmission function (OTF) for SiO_2_/Al films with variant thickness by RCWA and EMT. Middle and bottom panels are the real and imaginary part of *k_z_* for variant *k_x_* calculated by Bloch theorem without considering the Al absorption. OTF plots in logarithm scale as function of (**b**) unit thickness *d* and (**c**) metal film filling factor *f*. Reproduced from [37]. Copyright © 2015, WILEY.

The rigorous calculation of BPPs mode could be performed by rigorous coupled wave (RCWA) by finding eigen values of the transmission matrix. As shown in Figure 6a, the finite thickness of SiO_2_/Al films bend the equi-frequency contour, forming a specified *k_x_* wave vector range for propagation BPPs modes. The upper *k_x_* limit is determined by the Brillouin zone edge with *k_z_* = π/*d*, where *d* is the total thickness of a pair of SiO_2_/Al films. Correspondingly, the upper boundary of the calculated OTF window, shown in Figure 6a (top panel), coincides with the *k_x_* limit depicted in the bottom panel. It is obvious that the relation curves of *k_x_* and *k_z_* calculated by RCWA do not coincide well with that by EMT, especially for large *k_x_*, which demonstrates that the EMT method could only be an approximate analysis of metal–dielectric systems.

On the other hand, the lower boundary (unlike the upper boundary) of the transmission window is not equal to the calculated Bloch modes’ lower *k_x_* limit. This point is attributed to the contribution of the two surface modes confined at the interface between the surrounding medium and hyperbolic structure with SiO_2_/Al films. Moreover, the OTF window positions could be tuned by variant film thickness as shown in the top panel of Figure 6a, where the reduction of SiO_2_/Al film thickness from 30 nm/15 nm to 20 nm/10 nm delivers considerable extension of the upper *k_x_* limit for Bloch modes. This point can be clearly seen in Figure 6b. Further, the change of SiO_2_ film filling factor would shift the lower OTF window boundary, shown in Figure 6c.

## 3. Plasmonic Interference Lithography

Laser interference lithography (LIL) is a popular nanofabrication method due to its maskless, low-cost, parallel and large-area features, and it has attracted a great deal of interest and shows promising application in manufacturing a great variety of functional structures with periodical nano patterns [45], like hologram gratings, nano pillar arrays, two dimensional (2D) and three-dimensional (3D) photonic crystal structures, biosensors, etc. Suffering from the diffraction limit, the half-pitch resolution of conventional LIL is theoretically restricted as one quarter of the light wavelength. Light sources with shorter wavelength, like DUV, EUV and even soft x-ray, could achieve much smaller interference patterns, but the expensive laser setups and complex processing hampers their practical applications for common researchers.

### 3.1. SPPs Interference Lithography

**Figure 7:**
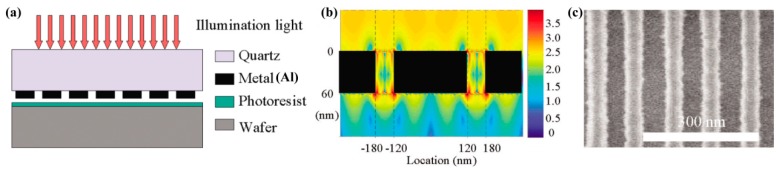
(**a**) Schematic structure of grating excited SPPs’ interference lithography; (**b**) Simulated electric field intensity distribution; (**c**) Resist recording results of SPPs’ interference fringes. Reproduced from [21], Copyright © 2004, AIP.

SPPs could be manipulated with a propagation wavelength much shorter than that of the same-frequency light in dielectric. This feature could be readily explored for subwavelength interference lithography. In 2004, aluminum grating with a 300 nm period and 50 nm wide slot was used to excite SPPs under 436 nm wavelength light, forming about 100 nm period interference lines inside resist beneath the grating (Figure 7) [21,46]. Further, a reflective cladding metal slab below the resist layer is proposed to generate high contrast interference fringes of coupled SPP waves [47,48].

**Figure 8:**
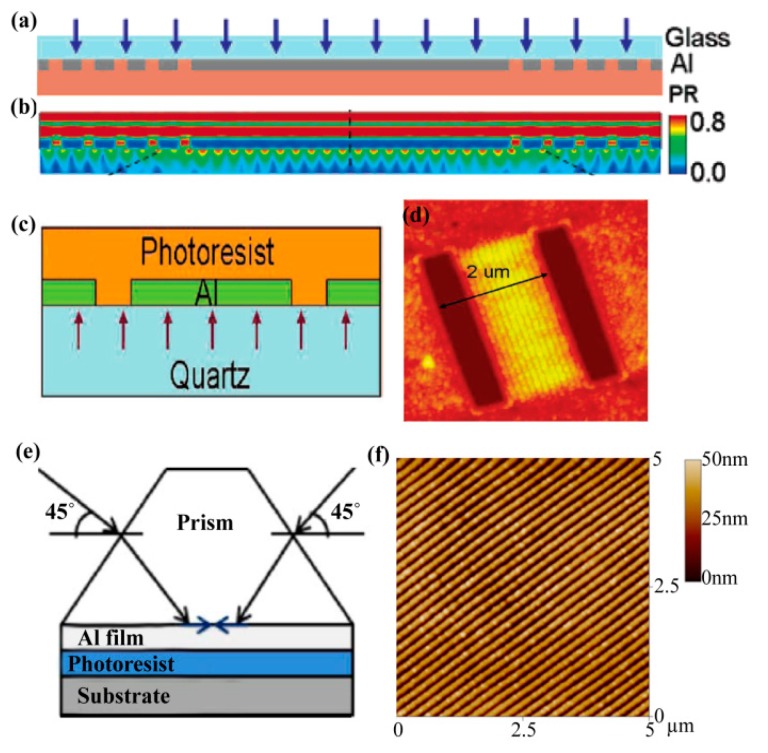
(**a**) Schematic configuration of SPPs’ interference lithography with two parallel gratings; (**b**) Cross sections of electrical field |*E*| distribution in (a); (**c**) Schematic experimental configuration of SPPs interference lithography; (**d**) Exposure pattern of structure (c); (**e**) SPPs’ interference through prism excitation; (**f**) Atomic force microscopy (AFM) image of one-dimensional interference patterns by prism. Reproduced from: (a,b), [49], Copyright © 2005, ACS; (c,d), [50], Copyright © 2009, ACS; (e,f) [52], Copyright © 2010, OAS.

To get SPP interference patterns beneath the grating structures with acceptable uniformity, the grating structure for SPPs’ excitation should be carefully designed to be compatible with the SPP mode. By imposing two or multiple gratings, SPPs’ interference beyond the diffraction limit could also be realized in the central region (Figure 8a–d) [49,50,51]. However, the interference pattern area size of this method is greatly restricted due to the inevitable great absorption and scattering of SPPs as they propagate along rough metal film.

SPPs’ prism excitation in the Kretschmann and Otto schemes could be introduced for large-area SPP interference lithography (Figure 8e,f) [52,53], but the resolution of the interference pattern is limited due to the finite refractive index of the prism material [54].

### 3.2. Odd SPPs Mode Interference Lithography

**Figure 9:**
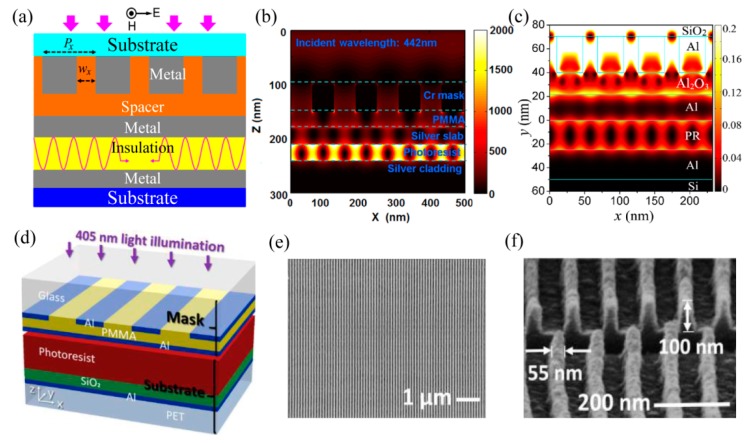
(**a**) Schematic structure of the odd SPP mode interference lithography; Simulation results at the wavelength of (**b**) 442 nm and (**c**) 193 nm; (**d**) Experimental structure for the odd SPP mode interference lithography; (**e**) Top view and (**f**) Cross section of SEM pictures of 100 nm-thick photoresist. Reproduced from: (b), [55], Copyright © 2009, Springer; (a,c), [56], Copyright © 2014, NPG; (d–f), [57], Copyright © 2016, ACS.

As shown in the Section 2.1, MIM structure helps to realize modification of SPP mode with much larger transverse wave vector *k_x_* and tailor the ratio between *E_x_* and *E_z_* components. This feature could be utilized for much smaller SPPs’ interference period by launching the odd SPP mode and improve pattern contrast by inhibiting the transverse electric field *E_x_* component (Figure 9a,b) [55]. The SPPs’ interference lithography using the odd mode in MIM structures is even extended to 193 nm DUV light and demonstrated numerically to push sub-diffraction resolution beyond 15 nm (Figure 9c) [56].

Recently, experimental demonstration shows that the 61 nm half-pitch resolution lines are obtained by the odd SPPs modes interference (Figure 9d–f) [57]. The odd modes are excited by the structure in the form of PMMA-Al-photoresist-SiO_2_-Al under the wavelength of 405 nm. Our experimental results to be published demonstrate SPPs’ interference lithography with SiO_2_-Al-photoresist-Al structure and 363.8 nm wavelength, yielding 45 nm half-pitch, 50 nm depth and area size up to 20 mm × 20 mm. Due to the strong resonance in the waveguide structure, the interference patterns exhibit large-area, high-aspect and high-resolution advantages.

### 3.3. BPPs Interference Lithography

**Figure 10:**
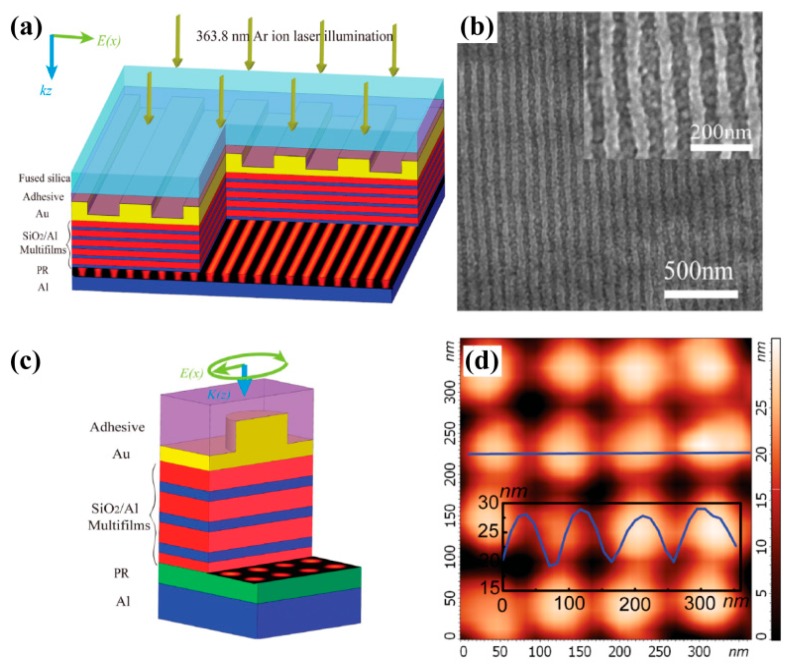
(**a**) Schematic configuration of two-BPPs’ interference; (**b**) SEM image of exposure photoresist; (**c**) Schematic configuration of four-BPPs’ interference; (**d**) AFM image of two-dimension interference pattern. Feature size of results is 45 nm. Reproduced from [37], Copyright © 2015, WILEY.

BPPs with a large wave vector propagate inside the specially designed bulk metamaterial with a hyperbolic dispersion relation. Some investigations were preformed to engineer BPPs and obtain deep subwavelength resolution interference patterns. An alternate metal/dielectric multilayer with the hyperbolic feature has been the preferred structure to render BPPs’ interference lithography. In the experimental demonstration, hyperbolic metamaterial composed of SiO_2_/Al films squeezes out BPPs generated by grating, and produces a large-area and uniform deep subwavelength pattern with half-pitch 45 nm (~λ/8) two and four BPPs’ interference (Figure 10). Much deeper resolution down to 22.5 nm (~λ/16) and a variety of BPPs’ interference patterns are feasible [37]. Recent simulation results exhibit sub-diffraction resolution of interference patterns, even reaching 16.5 nm under 193 nm illumination light [44].

### 3.4. Two-Surface Plasmon Polaritons Interference Lithography

Benefiting from the development of ultrafast laser, two-photon effects have been widely employed in many optic systems [58,59], such as two-photon absorption and two-photon polymerization for 3D micro fabrication and microscopes, etc. This non-linear optical response helps to get sharper point response function and improvement of imaging resolution in three dimensional spaces.

**Figure 11:**
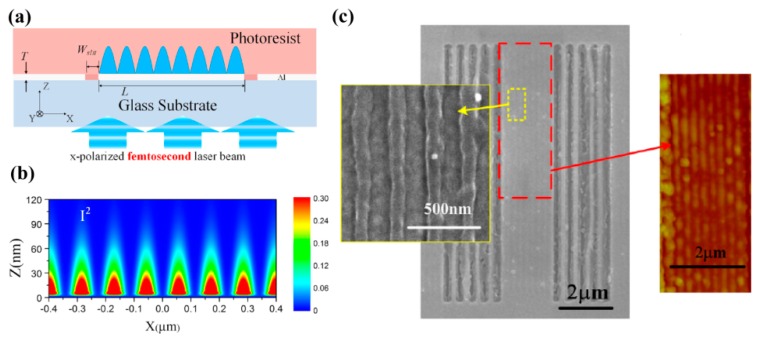
(**a**) Schematic structure of two-SPPs absorption interference lithography; (**b**) Simulated interference intensity distribution; (**c**) Experimental results of resist pattern. Reproduced from [60], Copyright © 2013, AIP.

Recently, this idea was extended to the case of two-SPPs’ absorption interference (TSPPA) lithography to improve the exposure pattern quality [60,61]. Just like two-photon absorption, two-SPPs’ quantums are simultaneously absorbed for the exposure of resist. So, the electric field intensity |*E*|^2^ response is replaced by |*E*|^4^ and a higher contrast or narrower SPPs’ hot spot response would be expected. At the same time the SPPs mode excited by photons helps to confine light within a small volume of spacer and increase the absorption of two SPPs. Huang et al. used two sets of side grating with 480 nm period and 800 nm femtosecond laser, and achieved 120 nm line width interference fringes by TSPPA lithography (Figure 11). Further, a similar experiment was performed with 400 nm wavelength femtosecond laser and 70 nm line width resist pattern was obtained.

### 3.5. Some Discussions

It is necessary and significant to present a comparison for the abovementioned plasmonic interference lithography with grating excited SPPs, prism excited SPPs, odd SPP mode, grating excited BPPs, as exhibited in Table 1.

It is possible to get a short wavelength SPP mode at the smooth bottom surface of partly etched Al-grating (second, third and fifth rows in Table 1). In fact, the idea of combining subwavelength grating with metal film for SPPs’ interference lithography has been investigated in a number of papers, mainly by numerical simulations. In contrast to the hyperbolic metamaterial BPPs’ interference structure (sixth row in Table 1), the obvious difference lies in the fact that the light field transmitted through the single Al film contains not only the excited SPP modes, but also those grating diffraction orders which are not matched. As a result of this point, some unwelcome aspects would be encountered both in design and practical processes for SPPs’ interference lithography. To avoid distortions from multiple diffraction orders, the designed gratings for ultra-short wavelength SPPs’ excitation are usually configured with a small subwavelength period or large period but nano features. As a result, costly equipment like FIB or EBL are usually used for grating fabrication. This, inevitably, greatly reduces the assumed advantage of sub-diffraction lithography and constrains the SPPs interference pattern area to tens of micrometers, considering the cost of FIB or EBL. Due to the above concerns, careful design of grating and the fabrication errors control of metal film structure would be required. Further, it would not be feasible for tuning the interference period with a fixed grating-metal film sample.

Fortunately, BPPs interference based on hyperbolic metamaterial plays a key role in generating large-area, uniform and deep subwavelength interference patterns with the ease of grating fabrication by conventional LIL. For alternate metal/dielectric multilayer coupling the BPP mode, the transmitted light is effectively restricted in specific *k_x_* wave vector window of hyperbolic metamaterial, outside of which the light transmission drops to zero. Benefiting from this point, a uniform pattern could be obtained by the interference behavior of purified plasmonic modes. The grating pitch could be several times that of interference fringes, allowing no need for EBL and FIB tools for grating fabrications and facilitating large-area and low-cost periodical patterns. Also, the flexibility for tuning the interference period and laser wavelength would be feasible within the transmission window, which will be demonstrated in our further work.

## 4. Plasmonic Direct Writing Lithography

Localized surface plasmons (LSPs) are employed in nano optical lithography associated with sharp metallic tips, nano metal particles and specifically designed nano apertures like bowtie structures, in which the large LSPs fields deliver nanoscale lithography resolution. Localized plasmonic lithography is usually performed in point-to-point writing manner, and it is easy to do in parallel by integrating multiple plasmonic lens over one head to increase throughput.

### 4.1. Localized Surface Plasmons Structures

Unlike SPPs and BPPs where light field is mainly confined to two-dimensional space or expanded into three-dimensional space, LSPs exists in a point-like region with zero dimensions. The typical LSPs mode structure is a nano metallic sphere, in which light illuminating it would excite free electrons oscillation inside sphere dipole resonator [62,63]. The strong resonance of light accompanying the ultra-small spacer effect yields a great field enhancement and could be directly used for nano lithography.

**Figure 12:**
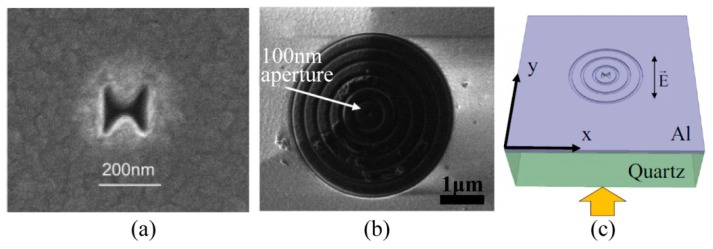
Various nanostructures used to confine SPPs and achieve a nano-scale spot. (**a**) Zoom in SEM picture of the bowtie aperture; (**b**) 100 nm aperture on the NSOM tip after the fabrication of the plasmonic lens; (**c**) Modified bowtie aperture with full circular grooves on the exit side of the film. Reproduced from: (a), [28], Copyright © 2006, ACS; (b), [67], Copyright © 2008, ACS; (c), [68], Copyright © 2011, AIP.

The simple way to obtain a light spot with subwavelength size could be done as light is leaked from a small hole. It, indeed, does its work in some cases like a metallic hole on the end of a fiber, usually employed in near-field scanning microscopes and used for scanning nanolithography as well [64,65]. One of the technical challenges of this method is the low transmission efficiency of light through nano holes, even in the case of employing metal coating. Another nano light localized structure is sharp metallic tips with ultra-small curvature, which behave like a lightning rod and facilitate in concentrating light energy around the tip apex. In this case, the light-enhancement factor should be large enough in comparison with the light illumination. LSP mode in some specially designed structure, like a bowtie (Figure 12a), helps to greatly enhance output light intensity and generates a nano hot spot [28,66]. In addition, by introducing a number of slits or grooves surrounding the LSPs’ hole or bowtie (Figure 12b,c) [67,68], excited plasmonic light energy would converge to the central part and is superimposed constructively to greatly enhance central focus intensity, as the slit/groove distances are well designed with proper SPP phase retardations.

### 4.2. Plsmonic Focusing Structures with SPPs and BPPs

**Figure 13:**
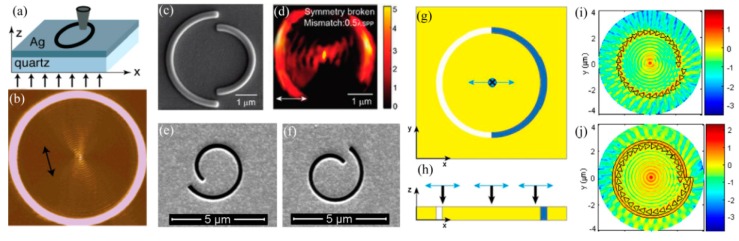
(**a**) Schematic structure of a circle cut into a 150 nm thick silver film; (**b**) Near-field pattern recorded by NSOM; (**c**) Symmetry broken plasmonic corral fabricated on the Au film; (**d**) |*E_z_*|^2^ distributions under linearly polarized lasers; A (**e**) left- and (**f**) right-handed spiral plasmonic lens in gold film; (**g**) Schematic geometry of the semicircular slits on the Au film filled with different medium; (**h**) A cross section of this structure; (**i**) A spiral triangle array structure; (**j**) Alternating spiral triangle array and spiral slot lens. Reproduced from: (a,b), [69], Copyright © 2005, ACS; (c,d), [70], Copyright © 2011, ACS; (e,f), [71], Copyright © 2010, ACS; (g,h), [72], Copyright © 2014, Springer; (i,j), [73], Copyright © 2012, OSA.

There are some plasmonic focusing structures without the requirement of an opaque confinement boundary, like metallic holes, just by superimposing constructively a great deal of SPP components at a focus point. The simple structure is a transparent ring on a metallic film, in which subwavelength focus appears at the ring center due to the convergence of SPP excited over the metallic ring (Figure 13a,b) [69]. Further, a two concentric half-ring structure with a radius difference of about half of the SPPs wavelength is proposed to generate a solid focus spot (Figure 13c,d) [70]. This method could also be done by using rings in a helix form (Figure 13c,f) [71]. SPPs focusing can be realized not only by changing the structural geometry but also by filling different medium into a semicircular slot. Based on the phase difference through changing the refractive index of the medium in the nano slits (Figure 13g,h), a tightly confined SPPs’ spot was achieved under linear polarization illumination [72]. In order to enhance the SPPs’ excitation efficiency, the spiral slot incorporating a spiral triangle array lens is designed and the spiral triangle structure could couple the azimuthal polarization component into SPPs (Figure 13i,j) [73,74].

**Figure 14:**
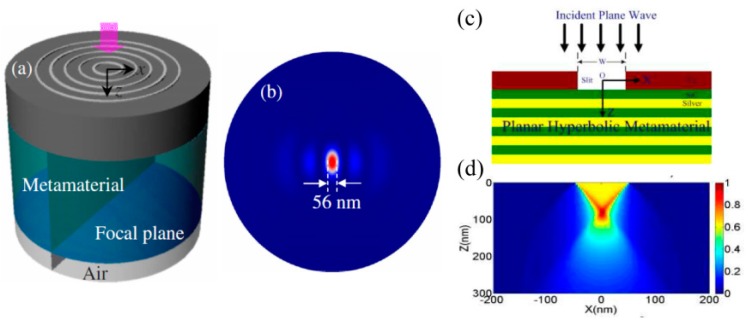
Nano spot achieved by focusing BPPs of hyperbolic metamaterial. (**a**) Metalens structure; (**b**) Simulation result of Metalens; (**c**) Plasmonic Fresnel plate structure; (**d**) Simulation result of plasmonic Fresnel plate. Reproduced from: (a,b), [75], Copyright © 2010, AIP; (c,d), [76], Copyright © 2010, OAS.

**Figure 15:**
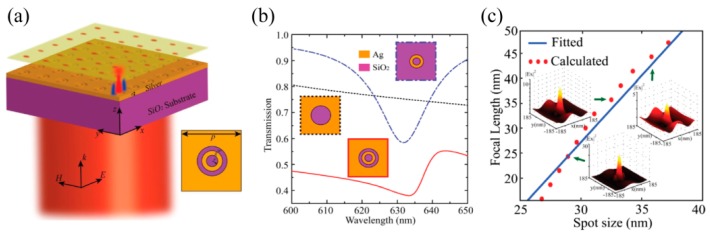
Nano focusing by plasmonic Fano resonance lens. (**a**) Schematic structure view; (**b**) transmission spectra; (**c**) nano focus length with variant focus size. Reproduced from [78], Copyright © 2016, RSC.

Particularly, hyperbolic metamaterial with multiple metal–dielectric films provides a volume space in which photons with large transverse wavevector *k_x_* propagate inside in the form of BPPs. Thus, a plasmonic Fresnel plate [75,76] and a hypergrating [77] structure could be designed for generating nano focusing effects inside or at the edge of BPP material by appropriately engineering BPP phases (Figure 14). Recently, an alternative nanofocusing approach using hybrid plasmonic Fano resonances in particular structures is proposed to achieve the subwavelength spot and elongate the focal length of focusing lens (Figure 15) [78].

### 4.3. LSPs Lithography with Nano Aperture

**Figure 16:**
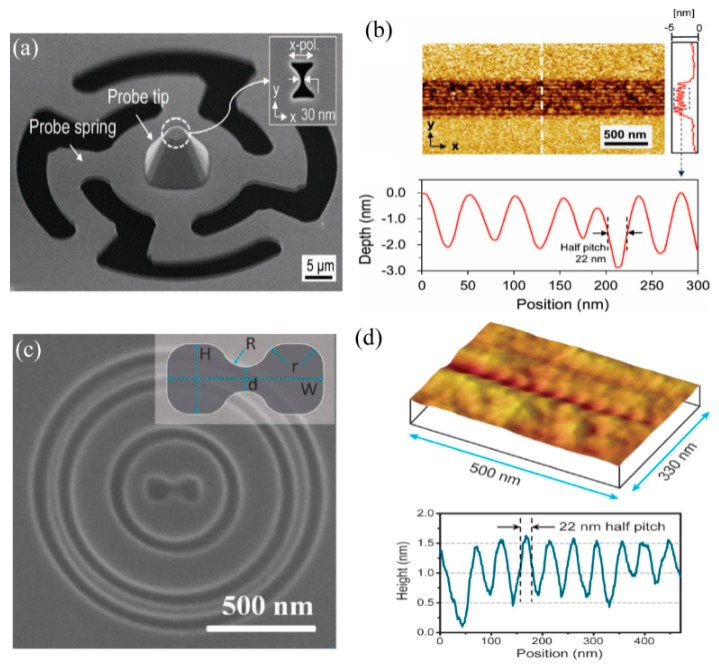
(**a**) SEM picture of circular contact probe with bowtie aperture for plasmonic lithography; (**b**) AFM image of resist pattern with half pitch 22 nm; (**c**) SEM picture of plasmonic lens consisting of a dumbbell-shaped aperture, a set of ring couplers (two inner rings) and a ring reflector (the outer ring); (**d**) Experimental results of resist pattern. Reproduced from: (a,b), [66], Copyright © 2012, WILEY; (c,d), [79], Copyright © 2011, NGP.

Some point-to-point scanning nano lithography methods are performed by the use of LSPs, including bowtie [28,66], metallic small hole probe [67] and bull eye structures [27], etc. The physical origin of nano optical spots for those methods is light resonance manipulation and confinement in nano spaces with specially designed LSPs or SPPs resonance nanostructures. Compared with the nano holes on a fiber end, one important improvement is the high efficiency of LSPs excitation. This occurs due to the partly opening geometrical feature of bowties and/or light concentrating into nano apertures with controlled surface plasmons on the boundary. Recent experimental reports show nano lithography with critical dimensions of about 30–80 nm and even 22 nm by combining SPPs, LSPs and the threshold effect of material under femo-second laser exposure (Figure 16).

**Figure 17:**
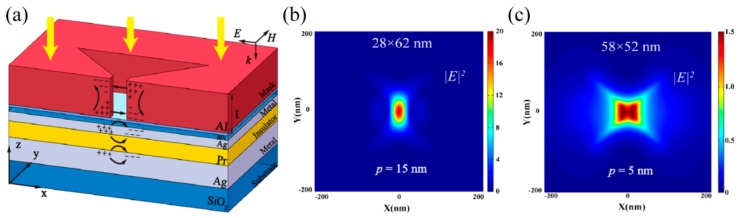
(**a**) Schematic structure of plasmonic lithography with bowtie aperture and plasmonic cavity lens resist recording structure; Simulation results (**b**) with cavity lens and (**c**) without cavity lens. Reproduced from [80], Copyright © 2015, Springer.

Further, some improvements have been proposed for bowtie lithography with considerable confinement of focus and enhanced exposure depth. By combining a plasmonic cavity lens with bowtie, it is demonstrated that both resolution and focus depth could be significantly enhanced (Figure 17) [80].

### 4.4. LSP Lithography with Apertureless Tips

**Figure 18:**
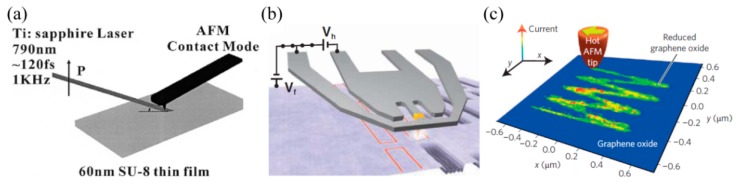
(**a**) Apertureless tip optical nanolithography; Scanning probe nanolithography based on (**b**) thermo and (**c**) chemical reactions. Reproduced from: (a), [81], Copyright © 2002, AIP; (b), [83], Copyright © 2010, AAAS; (c), [84], Copyright © 2014, NGP.

Usually, two issues associated with LSPs aperture structure deliver some inconveniences in their applications. Firstly, LSPs light is mainly confined in the transversal direction and emitted light from the apertures decays exponentially and becomes quickly emanative in the *z* direction. This inevitably results in considerably decreased focus depth and shallow feature profiles of the photoresist. The second concern is related to the strong dependence of light spot size on the nano-aperture geometrical shape. For instance, the partly opening aperture of a bowtie usually yields a non-circular light spot and hot points around the aperture edges. Apertureless metallic tips can be employed for optical fabrication in the nano dimension as well by the help of hot spot effect (Figure 18a) [81,82], thermo (Figure 18b) [83] and chemical (Figure 18c) [84] reactions around the metal tips. The hot spot usually delivers considerable enhancement of light concentrated at the tip apex. The other useful advantage of apertureless tips is the ease of fabrication through simple processes, including chemical etching. Unfortunately, spot enlargement and intensity decaying exists in this method as well.

**Figure 19:**
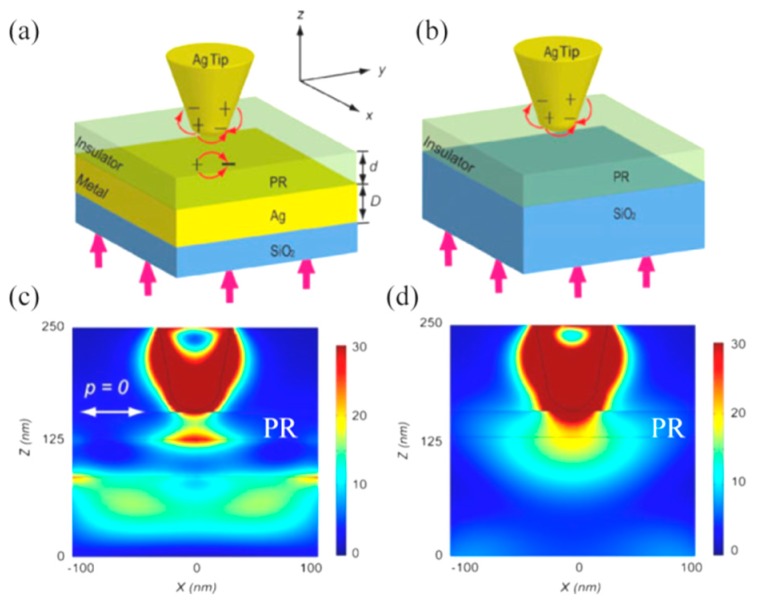
Schematic of nanolithography with (**a**) tip–insulator–metal and (**b**) tip–insulator structures; Simulated electric field intensity distribution of (**c**) tip–insulator–metal and (**d**) tip–insulator structures. Reproduced from [85], Copyright © 2013, Springer.

Those LSP hot spots usually behave like dipoles and show non-symmetrical spots with different lateral sizes in two orthogonal directions, especially at positions slightly away from bowtie structures. Some modified structure for apertureless tip lithography is proposed to solve the above problems, as shown in Figure 19a,b [85]. The tip, photoresist and metallic layer form a tip–insulator–metal (TIM) structure, in which a highly confined mode of plasmonic light both in the transversal and longitude directions occurs, with normal light illumination beneath the substrate. In addition to the resolution improvement, the spot intensity inside the photoresist along the longitude direction becomes nearly uniformly distributed and results in an elongated depth of focus. Numerical simulations show that full width at half maximum (FWHM) of the spot size could reach sub-10 nm by optimizing geometrical parameters of the TIM structure. Moreover, circularly polarized light illumination in the normal direction beneath the metal layer delivers a regular spot in a circular shape (Figure 19c,d).

### 4.5. Precise Control for Writing Head in Plasmonic Lithography

**Figure 20:**
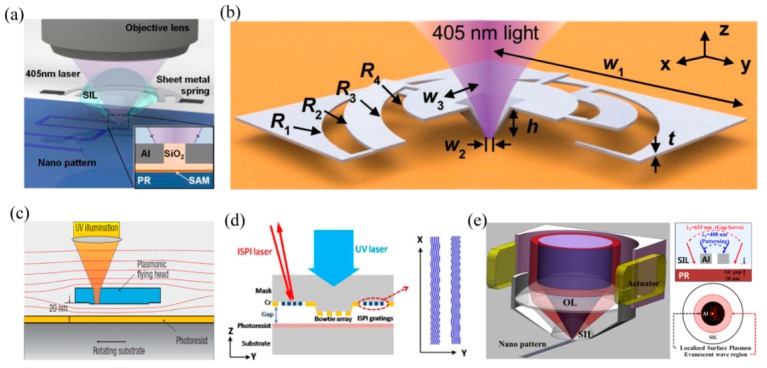
(**a**) Local plasmonic lithography system with a contact probe on a metal spring; (**b**) Schematic view of metal spring; (**c**) Cross-section schematic of the plasmonic head flying above the high-speed rotating substrate; (**d**) Schematic diagram of the nanolithography setup with ISPI gratings; (**e**) Schematic of the plasmonic optical head. Reproduced from (a), [86], Copyright © 2009, OSA; (b), [66], Copyright © 2012, WILEY; (c), [27], Copyright © 2008, NPG; (d), [87], Copyright © 2014, Springer; (e), [88], Copyright © 2012, AIP.

It is worth describing the efforts to overcome the major technique barrier of precisely controlling the plasmonic writing head in nano scanning lithography. As the LSPs’ focus is positioned in ultra-close proximity to the photoresist, usually in several and tens of nanometers, it is critical to hold on to the writing head in the scanning process with precise height, as well as its pitch and roll angles with respect to the wafer. In the flying plasmonic lens lithography experiment, an air-bearing surface used in magnetic storage disks is employed to generate a 20–100 nm air spacer between a rotating wafer with a high speed of ~10 m/s and a plasmonic lens, which is inscribed on a flying head designed by aero dynamics (Figure 20c) [27]. In some investigations, a sheet metal spring, on which the plasmonic writing head is held, is used to maintain a nearly stable posture of head during the scanning process (Figure 20b) [66], and the writing head is physically contacted with and scanned over a wafer with a speed of 10 mm/s (Figure 20a) [86]. In a passive control manner, a precise and online measurement of air working distance is performed by introducing the ISPI grating method (Figure 20d) [87] and an evanescent wave gap distance sensor (Figure 20e) [88]. The measured signal is used to control the head position and realize the nearly fixed air gap.

## 5. Plasmonic Lens Imaging Lithography

### 5.1. Superlens, Hyperlens and Negative Refraction Lens

**Figure 21:**
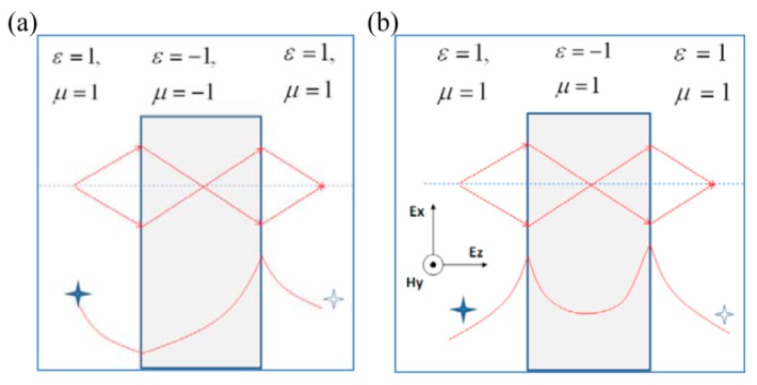
Imaging principle of (**a**) perfect lens and (**b**) superlens. Reproduced from [18]. Copyright © 2000, APS.

In 2000, Pendry proposed the concept of a perfect lens composed of a slab with a negative refractive index (NRI), in which all evanescent waves delivering subwavelength information could be amplified through the lens [18]. The amplification effect compensates the exponential decay feature of evanescent waves in free space and brings a perfect image with infinitely high resolution and free from any aberrations (Figure 21a). Although some NRI metamaterial based on split ring resonators, and metallic wires have been demonstrated in the microwave frequency range [89,90], it is a great challenge to make an NRI perfect lens in optics, considering the issues of fabrication and great light absorption in metal structures.

Fortunately, evanescent waves could be amplified as well by using a superlens of metal film with single negative permittivity, which occurs in the near field with quasi-static approximation and transverse magnetic polarization (Figure 21b). This behavior could be regarded as the SPPs excitation over a broadband spatial spectrum, especially for the case of permittivity matching the surrounding media, as indicated by Equation (1). Accordingly, evanescent waves would be coupled to the other side of a superlens with proper thickness.

**Figure 22:**
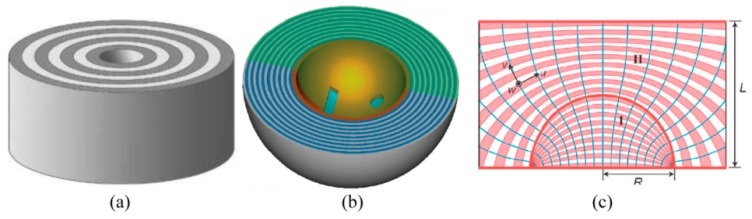
Schematic structures of variant hyperlenses with metal-dielectric films in (**a**) circular cylinder; (**b**) spherical form; (**c**) complex transformed coordinates. Reproduced from: (a), [91], Copyright © 2006, OAS; (b), [92], Copyright © 2010, NPG; (c), [93], Copyright © 2008, ACS.

A hyperlens is formed by introducing hyperbolic metamaterial to realize superresolution imaging in a magnifying or demagnifying manner. The commonly used structure of a hyperlens is multiple stacked metallic–dielectric films in concentric cylindrical or spherical form, as shown in Figure 22a,b. The BPPs inside the films help to transfer subwavelength information and form the relation of object and image between the inner and outer surface. By employing complex coordinate transformation, a planar hyperlens is also available with a flat surface for the input and output port (Figure 22c). In spite of the fascinating feature of zoomed imaging ability, hyperlenses do not seem appropriate as candidates for plasmonic lens lithography, because of their ultra-small imaging field size down to about several hundred nanometers, mainly due to the great loss and non-uniform image performances inside metamaterial films.

**Figure 23:**
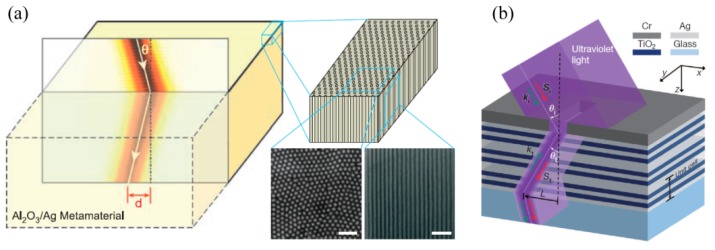
Optical bulk negative refraction index lens by (**a**) Silver nanowire metamaterials and (**b**) Stacked plasmonic waveguides metamaterial. Reproduced from: (a), [99], Copyright © 2008, AAAS; (b), [39], Copyright © 2013, NPG.

Some designed structures, like photonic crystals [94], usually show negative refraction behavior without the need for negative refraction index (NRI) materials. This occurs mainly due to the negative dispersion relation for some special optical modes. SPPs and BPPs behave in this way in some cases and are employed for NRI lenses. The NRI material in the optical range was experimentally demonstrated by pairs of parallel Au nano rods [95]. Subsequently, fishnet [96,97], metal–insulator–metal waveguide [98] and arrayed nano metallic wires [99] structures were investigated, which extends the illumination wavelength range from near-IR to visible. The most used NRI lens is a stack of hyperbolic structure [39], in which BPPs with hyperbolic dispersion contours bring the left-handed response under transverse magnetic polarization (Figure 23).

### 5.2. Superlens Lithography

**Figure 24:**
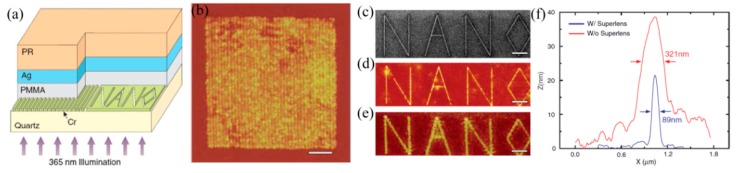
(**a**) Schematic of Ag superlens for plasmonic imaging lithography; (**b**) AFM image for 60 nm half-pitch feature through Ag superlens (scale bar, 1 mm); (**c**) The ‘‘NANO’’ object on the Cr film; AFM of the developed image on photoresist (**d**) with or (**e**) without a Ag superlens; (**f**) Cross section of letter ‘‘A’’ in (d,e), Scale bar in (c–e) is 2 mm. Reproduced from [22], Copyright © 2005, AAAS.

In 2000, Pendry firstly proposed the concept of a superlens as a simplified case of a perfect lens, in which evanescent waves could be amplified by a single negative permittivity slab [18]. The amplification effect delivers sub-diffraction limited imaging, which is subsequently experimentally demonstrated with a silver film [22,100]. The representative work reported in 2005, in which 60 nm half-pitch dense grating pattern was imaged in lithography resist by utilizing a 35 nm Ag film, 40 nm PMMA spacer between the mask and superlens, and a 365 nm Hg lamp light [22]. Also a “NANO” characters pattern with 89 nm line width was presented to demonstrate the superlens’ ability for complex patterns (Figure 24).

**Figure 25:**
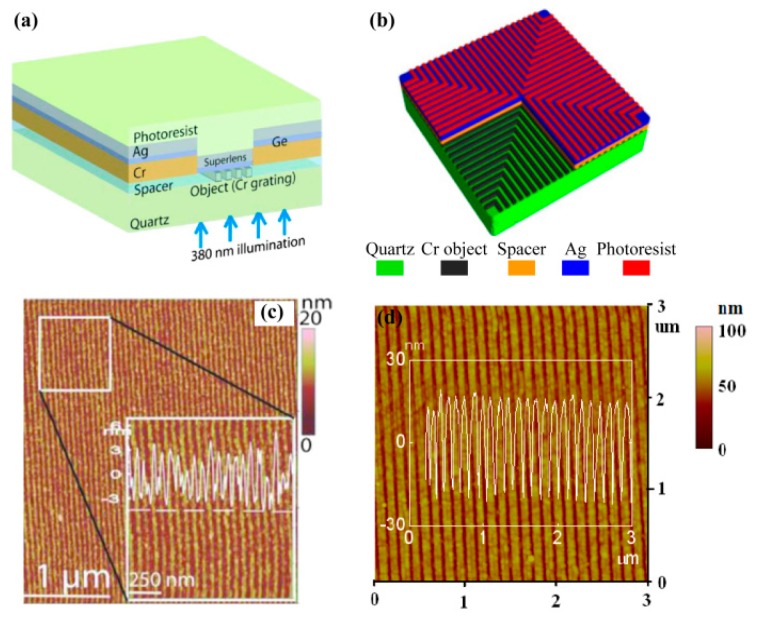
(**a**,**b**) are the schematic structures of a superlens; AFM image of resist pattern with half-pitch (**c**) 30 nm and (**b**) 60 nm. Reproduced from: (a,c) [101], Copyright © 2010, AIP; (b,d) [103], Copyright © 2012, ACS.

To improve the resolution and performance of superlens lithography, further reported investigations mainly concentrated on the aspects of geometrical parameters optimization, reducing films roughness and damping loss inside, etc. Chaturvedi et al. employed nano imprinting and Ge seeding technology to form a 6 nm spacer and a 15 nm smooth Ag superlens, and observed a 30 nm half-pitch resolution result, in spite of the extra attenuation caused by germanium (Figure 25a,c) [101]. Liu et al. performed the experimental and analytical demonstration, which showed that a smooth interface was a key factor for realizing a high-performance superlens [102]. However, damp losses become dominant in determining imaging resolution while the roughness is reduced to less than 2 nm. They also performed superlens lithography without a Ge/Ni wetting layer, and realized a 50 nm half-pitch and much higher aspect ratio with ~45 nm resist thickness (Figure 25b,d) [103].

At the same time, the enhancement of subwavelength imaging has been theoretically and experimentally investigated by inducing adequate roughness and/or loss [104,105,106]. Bagley et al. verified enhancement of image with subwavelength roughness interface beyond that of a smooth film lens by the method of moments and the T-matrix method [107]. Wang et al. combined surface sinusoidal roughness with the additional loss and achieved flatter transfer function and 86% reduction of beam width compared to the lossless flat superlens [108]. Huang et al. demonstrated that surface roughness can damp resonance peaks as well as the near-field interference by reducing the transverse propagation length of SPPs in a spatial domain [109]. In our recent work, further investigation was made to elucidate the paradox conclusion about the roughness and loss issue [110]. It is found that, surface roughness mainly affects the imaging quality (e.g., uniformity, deviation), while the loss coefficient mainly determines the imaging resolution. In spite of more transmission of high spatial frequency components with a rough surface, the interferential noise among dense-distributed images is becoming more severe with increasing roughness. With the coexistence of both roughness and loss, based on our investigation, retaining adequate loss is an effective way to improve the performance of imaging, which would greatly abate the resolution enhancement by loss reduction.

### 5.3. Plasmonic Reflective Lens Lithography

**Figure 26:**
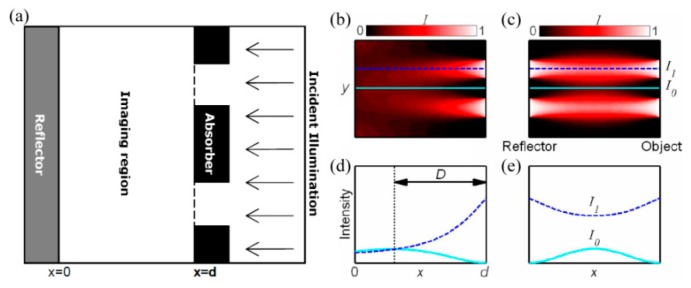
(**a**) Schematic structure of plasmonic reflective lens lithography; Image intensity distribution (**b**) without reflection and (**c**) with reflection. The longitudinal profile distribution of *I*_0_ and *I*_1_ in (b,c) are shown in (**d**,**e**). Reproduced from: [111], Copyright © 2007, OAS.

Near-field lithography methods are usually constrained in practical applications due to the poor quality of resist patterns characterized with shallow profiles, low contrast and great aberrations compared with masks. The decaying feature of evanescent waves is believed to be the dominating reason for this issue. Arnold et al. proposed theoretically that a plasmonic lens would enhance evanescent waves in a reflection manner and help to relieve this effect (Figure 26) [111].

**Figure 27:**
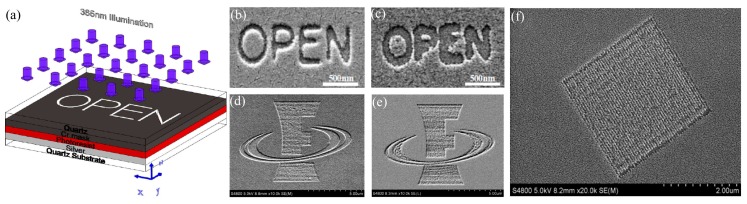
(**a**) Schematic structure of plasmonic reflective lens lithography; (**b**,**d**) Plasmonic reflective lens lithography imaging and (**c,e**) conventional near-field imaging lithography. Feature size of the pattern is approximately 50 nm. (**f**) SEM picture of 32 nm half-pitch pattern for reflective plasmonic lens lithography. Reproduced from: [23], Copyright © 2013, OAS.

By using a plasmonic reflective lens, deep sub-diffraction imaging lithography is numerically and experimentally demonstrated by imaging nano letters with about 50 nm line width and dense lines with 32 nm half-pitch resolution (about 1/12 wavelength), as shown in Figure 27. Compared with the control experiment without a plasmonic reflective lens, resolution, contrast and depth of imaged resist patterns are remarkably improved, especially for isolated nano features [23]. In conventional lithography, reflection from the layer beneath the resist is not welcome, to avoid any standing wave effect. In SPP lithography, however, the near-field space inside the resist hampers any standing wave effect, and full manipulation of evanescent waves in the form of SPPs dominates the lithography pattern quality.

### 5.4. Plasmonic Cavity Lens Lithography

The combination of a superlens and plasmonic reflective lens would deliver further resolution enhancement of imaging lithography, as demonstrated both in simulation and experiment. The opening space, as shown in a superlens and reflective lens, results in the amplification of evanescent waves only in one side and could not compensate the waves decaying in the other direction. Thus, two unwelcome features are encountered, especially in the superlens scheme. One is the ultra-shallow exposure depth, less than 10 nm, and the other is the low contrast of images due to the great decay of evanescent waves and the *E_z_* component’s negative contributions.

**Figure 28:**
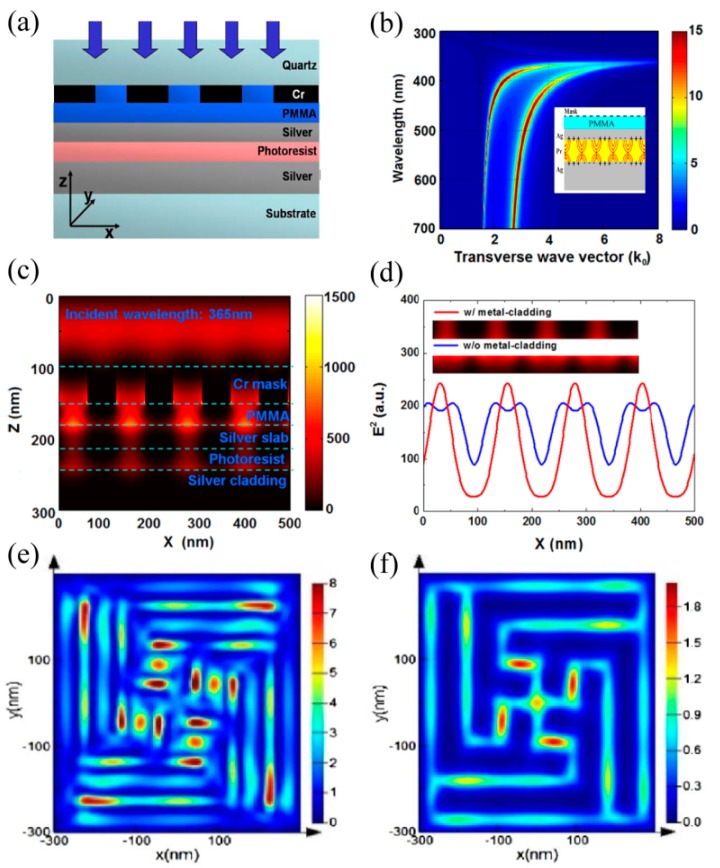
(**a**) Schematic structure of plasmonic cavity lens lithography; (**b**) H-field amplitude at the interface of the Ag superlens and photoresist as a function of the optical wavelength and the transverse wavevector through the plasmonic cavity lens structure, inset is the approximately mirror-symmetric distribution of surface charges; Simulation results of the object with (**c**,**d**) 62 nm and (**e**,**f**) 45 nm resolution. Reproduced from: (a–d), [55], Copyright © 2009, Springer; (e,f), [112], Copyright © 2013, OSA.

The plasmonic cavity lens, regarded as a metal-cladding superlens structure, could significantly amplify evanescent waves in the entire photoresist region due to the SPPs multiple reflections inside the cavity (Figure 28a,b) [55]. Fringes with about 62 nm half-pitch (about 1/13 wavelength of incident light) can be obtained in numerical analysis with greatly improved intensity contrast (Figure 28c,d). Also, demonstrated numerically, is the fidelity improvement together with the contrast increase in plasmonic cavity lens lithography (Figure 28e,f). Further, 15 nm resolution could be realized if the resist layer of the plasmonic cavity lens is reduced to 10 nm [112].

**Figure 29:**
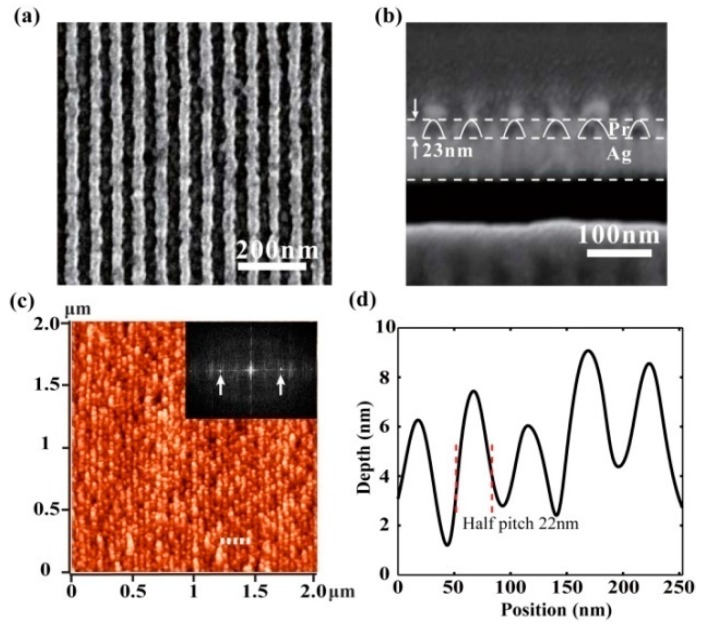
(**a**,**b**) are the scanning electron microscope (SEM) pictures and cross profile of the resist pattern for half-pitch 32 nm, respectively; (**c**,**d**) are for half-pitch 22 nm. Inset in (c) is Fourier spectrum distribution. Reproduced from [24], Copyright © 2015, AIP.

In our recent work, 32 nm and 22 nm half-pitch resist patterns were obtained in a plasmonic cavity lens lithography experiment under 365nm light wavelength [24]. The plasmonic cavity lens structure is in the form of a 20 nm thick Ag layer–30 nm thick Pr layer-50 nm thick Ag layer and the exposure depth in the Pr region is 23 nm, nearly 4 times that of the superlens (Figure 29a,b). The depth increase of the Pr recording pattern, with the help of the multiple resist layers etching process, brings a high aspect ratio of nano patterns, reaching about 2.5. The minimum resolvable half-pitch resolution, down to 22 nm, has already been experimentally demonstrated by the abovementioned plasmonic cavity lens (Figure 29c,d).

### 5.5. Resolution Enhancement Method for Plasmonic Lens Lithography

Further improving the resolution of the plasmonic lens is a challenge due to the finite amplification ability for the evanescent wave. In addition, the associated concern of a high-quality lithography pattern also needs to be resolved. Fortunately, resolution enhancement techniques in conventional photolithography may be referred to as one solution.

**Figure 30:**
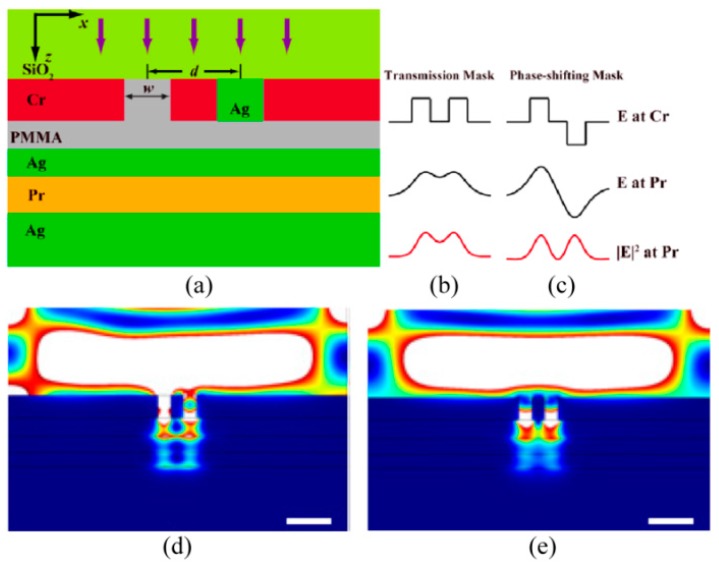
(**a**) Schematic structure of plasmonic lithography incorporating PSM; (**b**,**c**) Principle of plasmonic PSM; Simulation results of 30 nm resolution pattern (**d**) with PSM and (**e**) without PSM. Reproduced from [113], Copyright © 2011, OSA.

In 2011, we proposed the plasmonic phase-shifting mask (PSM) method and neighboring slot being filled by Ag and PMMA to induce the π-phase shift with the same transmittance amplitude [113]. The simulation result shows the resolution of plasmonic lithography is pushed to 30 nm with 30 nm exposure resist depth by the designed plasmonic PSM structure (Figure 30).

On the other hand, to significantly amplify the evanescent waves by SPPs modes, the plasmonic lens is usually used in the near-field region, where evanescent waves could contribute to the subwavelength resolution imaging. This, inevitably, delivers a very short working distance to the plasmonic lens, much smaller than the light wavelength. The higher resolution plasmonic lens has, the shorter working distance it exhibits. Suffering from the short working distance feature, the plasmonic lenses in experiments with deep subwavelength resolution are usually contacted with objects and/or recording medium, like mask and photoresist in superlens lithography, etc. The abovementioned works demonstrate the scheme of intimate contact between the plasmonic lens and the photoresist, which leads to the issue of recycling, using the small foot-print in lithography application. Besides, some of those methods seem complicated and challengeable for practical fabrication. This concern imposes great obstacles and challenges for the practical applications in lithography, microscopy and optical recording, etc. A new resolution enhancement technique that improves the image contrast and achieves non-contacted plasmonic lens application is more desirable. Moreover, it is demonstrated recently that the nano air gap distance (about 20–200 nm) of the plasmonic lens could be measured and controlled precisely with the help of some novel techniques, such as the air-bearing surface [27], the interferometric spatial phase imaging system (ISPI) [87], and the nano gap servo system [88], etc., indicating the possible access to non-contacted plasmonic lens applications and the requirement and significance of elongating the plasmonic lens’ air working distance.

**Figure 31:**
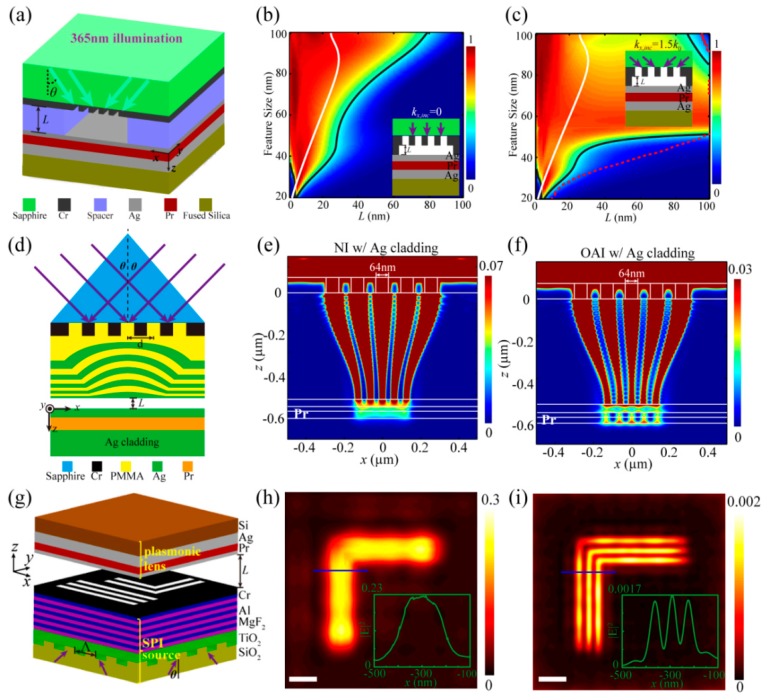
(**a**) Schematic structure of SPPs cavity lens imaging under off-axis illumination (OAI); Imaging contrast of dense line pattern as a function of air working distance and feature size in the case of (**b**) conventional near-field imaging and (**c**) plasmonic cavity lens with OAI (illumination light *k_x_*_,inc_ = 1.5*k*_0_); (**d**) Schematic structure of planar hyperlens imaging under OAI; (**e**,**f**) are the simulation results of the structure in (d) under normal and off-axis illumination, respectively; (**g**) Schematic structure of plasmonic cavity lens imaging under surface plasmon illumination (SPI); (**h**,**i**) are simulated imaging results of the L-shape feature under normal and off-axis illumination, respectively. The air working distance in (e,f,h,i) is 40 nm. Reproduced from: (a–c), [25], Copyright © 2015, NGP; (d–f), [114], Copyright © 2014, Springer; (g–i), [115], Copyright © 2015, Springer.

Conventional off-axis illumination (OAI) is widely used to improve resolution and depth of focus in projecting optics lithography. The SPI method would push the illumination wave vector from propagating wave to evanescent wave, and shows the capability of improvement of resolution and elongation of air working distance. In our work, surface plasmon illumination (SPI), which means that the off-axis illumination wavevector is pushed to the evanescent wave, is proposed to elongate the air working distance of a “near-sighted” plasmonic lens [25,114,115,116]. The method is based on an SPI source excited by the prism or the bulk metamaterial and a plasmonic cavity lens in the form of an MIM structure. Numerical simulations concentrate on the imaging performance of a plasmonic cavity lens and a planar hyperlens, and demonstrate the air working distance is remarkably elongated in the resolution range from 30 to 100 nm (Figure 31). On the other hand, the modulation effect of electric field components in the Pr region plays a necessary role due to the inference behavior of diffraction lights from the deep subwavelength resolution pattern. It is believed that the proposed method helps to give a potential way for non-contacted plasmonic lens applications in lithography, microscopy and optical storage, etc.

**Figure 32:**
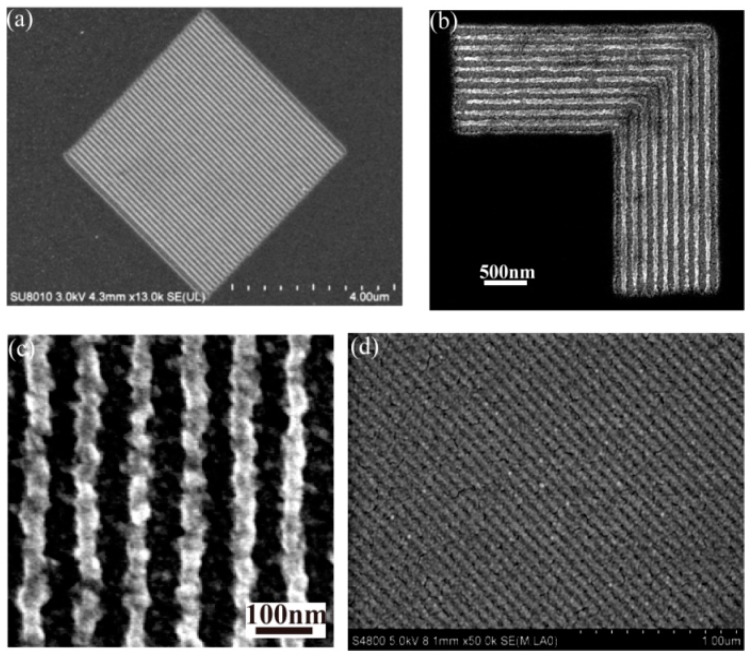
SEM picture of (**a**) 60 nm resolution with 120 nm air working distance under *k_x_*_,inc_ = 1.5*k*_0_, (**b**) 50 nm resolution with 50 nm air working distance under *k_x_*_,inc_ = 1.5*k*_0_, (**c**) 45 nm resolution with 20nm air working distance under *k_x_*_,inc_ = 1.5*k*_0_, (**d**) 32 nm resolution with 40 nm air working distance under *k_x_*_,inc_ = 2.5*k*_0_. Reproduced from [25], Copyright © 2015, NGP.

Further, the lithography experiments are rendered to establish the air working distance elongation effect. The air working distance is controlled in experiments by fabricating the Cr spacer around the mask pattern and air is the separation medium between the pattern and the plasmonic cavity lens. The photoresist recording results are shown in Figure 32, where the resolvable pattern contains the 60 nm half-pitch dense lines [25], 50 nm feature-size L-shape lines, 45 nm half-pitch dense lines and 32 nm half-pitch dense lines.

## 6. Process Control and Patterns Transfer of Functional Structures

As an optical lithography method, plasmonic lithography exhibits some common shortcomings, which are same as those occurrences in conventional proximity and projecting lithography, such as near-field proximity effect, shallow aspect depth for high resolution pattern, etc. Some solutions in conventional lithography could be applied to resolve the problems proposed in plasmonic lithography, as partly demonstrated in the following investigations.

### 6.1. Near-Field Proximity Corrections of Plasmonic Lens Lithography

Resembling that in projection optical lithography, resist patterns in plasmonic lithography usually show some proximity aberrations like broadened and shortened lines pattern, distortions of rectangular pattern shape, etc., which mainly arise from the limited resolution of the plasmonic lens and variant optical aberrations in the imaging process, as well. To relieve this concern and improve the fidelity of image patterns to some extent, some near field proximity corrections could be done in the optimization of masks in plasmonic lithography.

**Figure 33:**
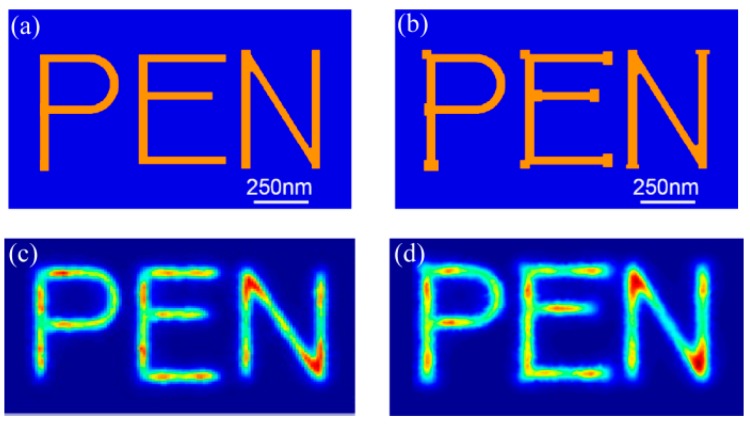
(**a**) Schematic of pattern on mask; (**b**) Optical proximity corrected mask design; Simulation results (**c**) before OPC and (**d**) after OPC. Reproduced from [23], Copyright © 2013, OSA.

Further numerical simulations show that optical proximity corrections of mask patterns help to improve image fidelity of two-dimensional nano patterns in plasmonic lens imaging lithography [23]. As shown in Figure 33c, the line end shortening occurs in letter “P” and “N” and the corner rounding effect in “P”, “E”, and “N”. The simply corrected mask nano “PEN” patterns and the calculated image light distribution with modified mask are presented in Figure 33b,d with improved shortening and the corner rounding effects. It is believed that further complicated and careful masks design, with adjacent grooves and lines, etc., would bring considerably improved fidelity of the imaging light pattern.

### 6.2. High Aspect Ratio Pattern Transfer by Multiple Layers

**Figure 34:**
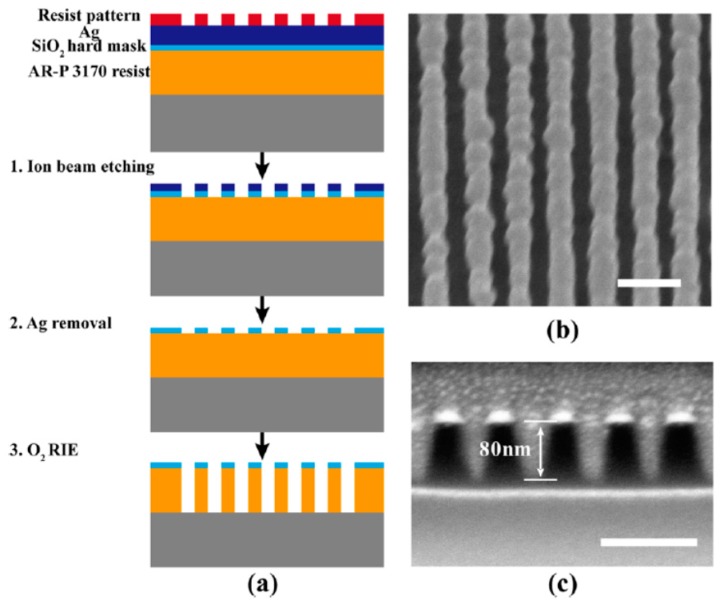
(**a**) Schematic diagram of the resist pattern transfer for plasmonic lithography. SEM pictures of half-pitch 32 nm bottom layer resist pattern (**b**) top view and (**c**) cross sectional view. Scale bar in (b,c), 100 nm. Reproduced from [24], Copyright © 2015, AIP.

Although the resist pattern depth inside the photoresist of plasmonic lithography is improved, it is not always deep enough for further transferring to bottom functional layers, usually with a thickness ranging from tens to hundreds of nanometers. Similar problems occur in commercial advanced lithography with ultra-deep resolution and greatly reduced short focus depth, like 193 nm lithography. In this case, the multiple resist layers technique helps to realize pattern transfer to deep patterns. As one example of this method, about 25 nm resist depth is obtained in the plasmonic cavity lens lithography structure for 32 nm half-pitch dense lines in the case of single exposure. However, about 80 nm thick resist lines pattern with a half-pitch of 32 nm would be achieved by subsequently two-step ion etching (Figure 34): (1) from top resist to a hard SiO_2_ layer; (2) from SiO_2_ hard mask to another resist layer which is not sensitive to lithography exposure [24]. It is a somewhat complex operation, but worthy for lithography resolution down to 32 nm with a low-cost lithography setup. The enhanced resist depth in the plasmonic lithography step should not be undervalued, as it is hard to transfer resist patterns with several nanometers thickness by the etching process. Moreover, the developed resist pattern accompanied with extra hard protection film may be used as the template for further nanoimprinting. On the other hand, the resist pattern could be directly transferred to the bottom Al film and materials below by dry etching.

### 6.3. Fabrications of Functional Structures via Plasmonic Lithography

**Figure 35:**
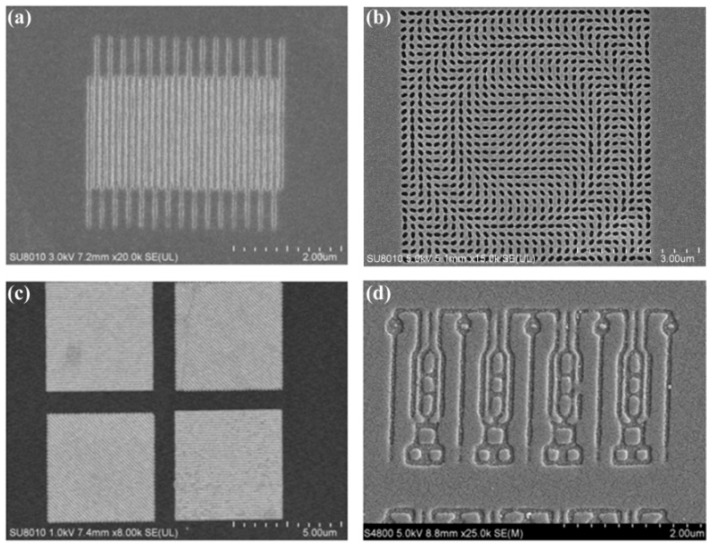
SEM pictures of plasmonic lithography for nanostructure employed in (**a**) integrated circuit, (**b**) nanofocusing optical lens based on metasurface, (**c**) surface plasmon polaritons couplers, (**d**) data recording circuit. Feature size of patterns ranges from 30 to 60 nm. Reproduced from [117], Copyright © 2015, AIP.

Due to the rapid advance of plasmonic lithography, some functional structures with a definite design have been successfully fabricated by plasmonic reflective lens lithography, accompanied by some extra etching processes. Shown in Figure 35b is an SEM picture of a fabricated Ag metalens composed of a great number of nano slots with a size of 65 nm × 150 nm and variant orientations, which help to focus light into a small region with opposite polarization relative to the incident light [117]. Also obtained in our investigations, is included a nano polarizer (Figure 35c), nearly random patterns resembling logical integrated circuits (Figure 35a,d), and a logo pattern of our institute (Figure 27d) [23], etc. These demonstrations, undoubtedly, show the great compatibility of plasmonic lithography patterns and the promising and potential applications of plasmonic lithography in a variety of nano structures like holograms, vortex phase plates, bio-sensors and solar cells, etc.

## 7. Conclusions and Outlooks

In a brief summary, the sub-diffraction feature of SPPs with a short propagation wavelength enables the design of a great variety of plasmonic structures, artificial materials and the plasmonic lens, and helps to realize optical lithography far surpassing the conventional diffraction limit of resolution without contributions from some additional manipulations like non-linear optic materials, threshold effect and double exposure, etc. Thus, the three types of plasmonic states—SPPs, BPPs and LSPs—exhibit different behaviors with respect to their propagation features, spatial distributions of modes, dispersion relations and electric field components, etc., and form the basics of engineering light in subwavelength scales and designing functional structures for interference, direct writing and imaging beyond the diffraction limit. The novel concepts, like the perfect lens, superlens, hyperlens, etc., were proposed and extensively investigated both in simulations and experiments, and their potential applications in nanolithography were demonstrated as well.

A great deal of achievements has been made in recent years, with the motivation for the improvement of resolving ability, fidelity, control and feasibility in plasmonic nano lithography. For early investigations, most research works are mainly focused on the demonstrations of the sub-diffraction ability of plasmonic lithography, in the form of SPPs interference structures, the superlens, LSPs structures like nano metallic particles and the bowtie, etc. Further, due to the concerns about the limited resolution, area size, low aspect ratio of plasmonic lithography patterns, fixed 1:1 transfer factor and physical contacting manner, etc., a great variety of methods from the aspects of plasmonic lens design, fabrication and process optimization and resolution enhancement were proposed and illustrated in experiments, including coupled SPPs mode interference in MIM structures, BPPs interference with demagnification factor, the plasmonic reflective lens, the plasmonic cavity lens and structured light illumination, the smooth plasmonic lens with doping and seeding films deposition methods, optimization of resist processing and combination with threshold effect, multiple resist layers, etc. Usually, those improvements rely on methods dealing with masks with appropriate illumination, pattern optimization, and reduction of negative factors associated with plasmonic lens structures, especially for light scattering and absorption in metal films. Furthermore, manipulations of imaging space, especially in the form of metal–dielectric–metal cavity structure, help to get much higher contrast, imaging fidelity and depth. Some auxiliary control techniques are employed, and deliver non-contacting, large-area and parallel plasmonic lithography accompanied by the help of the plasmonic lens design, like the flying plasmonic lens and some nano air gap measurement methods, relieving to some extent the practical application concerns of the physical abrasions of masks, precise metrology and efficiency, etc. Benefiting from those achievements, half-pitch resolution down to 32, 22, 16 nm and even below has been achieved in investigations, and the thickness of resist patterns was extended from a few nanometers to over one hundred nanometers, and pattern size in one sample to several millimeters and even centimeters. Some functional structures, like metasurface lens and polarizers, have been successfully fabricated by the plasmonic lithography method.

Generally speaking, extra efforts have to be taken for the further development of plasmonic lithography. In comparison with traditional lithography, the compatibility, qualities, controlling ability and infrastructures of plasmonic lithography have not been fully built yet. Some points should be particularly emphasized and require further systematic investigations, like a non-contacting plasmonic lens, reliable control of plasmonic lens leveling and focusing methods, convenient plasmonic lithography and following the transfer process with wide latitudes, uniformity of control of large-area patterns, etc.

## Figures and Tables

**Table 1:** Comparison of various plasmonic interference lithography technologies.

Methods and Features	Light Source & Wavelength	Excitation SPPs Structure	Excitation Structure Fabrication	Interference Pattern Period	Interference Pattern Area Size	Reference
**Grating-excited SPPs**	g-line Hg lamp & 436 nm	Grating Period 300 nm Slit width 50 nm	Electric beam lithography	100 nm	-	[21]
**Grating-excited SPPs**	i-line Hg lamp & 365 nm	Edge slit Slit width 200 nm	Focusing ion beam milling	120 nm	2 μm	[50]
**Prism-excited SPPs**	Ar ion laser & 363.8 nm	Prism	-	172 nm	20 mm × 20 mm	[52]
**Odd SPPs mode**	diode laser & 405 nm	Grating Period 245 nm Slit width 122 nm	Nano imprint	122.5 nm	10 mm × 10 mm	[57]
**Grating excited BPPs**	Ar ion laser & 363.8 nm	Grating Period 360 nm Slit width 180 nm	Laser interference lithography	90 nm	20 mm × 20 mm	[37]
